# Design Recommendations for Virtual Reality–Based Upper Limb Exercises From People With Tetraplegia and Spinal Cord Injury Rehabilitation Specialists: Focus Group Study

**DOI:** 10.2196/66832

**Published:** 2026-02-11

**Authors:** Andrew Goodsell, Mariel Purcell, Matthieu Poyade, Louise Cownie, Co-designers, Lorna Paul

**Affiliations:** 1 School of Health and Life Sciences Glasgow Caledonian University Glasgow United Kingdom; 2 Queen Elizabeth National Spinal Injuries Unit NHS Greater Glasgow and Clyde Glasgow United Kingdom; 3 School of Innovation & Technology Glasgow School of Art Glasgow United Kingdom; 4 Spinal Injuries Scotland Glasgow United Kingdom

**Keywords:** acute/sub-acute tetraplegia, co-design, focus groups, secondary care, spinal cord injury, upper limb rehabilitation, virtual reality

## Abstract

**Background:**

The global incidence of spinal cord injury (SCI) is between 10 and 80 new cases per million people each year. This equates to between 250,000 and 500,000 injuries worldwide per year. In the United Kingdom, approximately 4400 people per year sustain an SCI. People with tetraplegia report upper limb function as their highest priority for improvement after SCI. Using immersive virtual reality (VR) headsets, physical rehabilitation exercises can be completed in engaging digital environments. Immersive VR therefore has the potential to increase the amount of therapy undertaken, leading to improvements in arm and hand function. There is little evidence supporting immersive VR as exercise in SCI, especially while patients with SCI are undergoing acute rehabilitation. In SCI research, co-design of new interventions is not a widely adopted approach, yet people with tetraplegia want to contribute with their expert knowledge on their experiences of SCI.

**Objective:**

This study aims to explore the lived experiences of people with tetraplegia and specialist SCI therapists related to acute upper limb rehabilitation and identify design considerations for VR-based interventions targeting the upper limb.

**Methods:**

We conducted 7 online focus groups using Microsoft Teams: 4 with people with tetraplegia (n=15; age range, 36-65 years) and 3 with occupational therapists and physiotherapists specializing in SCI rehabilitation (n=11). Participants were asked to discuss their experiences and expertise about acute SCI upper limb rehabilitation and their opinions and ideas on the use of VR for upper limb rehabilitation. The transcripts were analyzed using content analysis, enabling the proposition of design characteristics of a VR-based intervention for upper limb exercise.

**Results:**

The study identified 5 major themes describing the clinical features, treatment, and recovery of people with SCI during the acute stage of SCI, their motivations for participating in therapy, and suggestions for the design of a VR intervention in treating the upper limbs following SCI.

**Conclusions:**

The themes identified in this study allow the elicitation of software requirements for a bespoke immersive VR platform for upper limb rehabilitation following SCI. They can also contribute to a better understanding of the advantages of using VR as an adjunct to upper limb rehabilitation. Additionally, participants used their expertise to suggest factors that would enable the development of a usable and effective intervention, as well as identifying potential pitfalls and software features to avoid during intervention development. These findings can be used to design accessible VR applications for use by people with tetraplegia and their therapists.

## Introduction

A spinal cord injury (SCI) results from an insult to the spinal cord and impacts nearly every aspect of a person’s life. People with SCI usually have a degree of permanent neurological disability, including muscular paralysis and impaired sensory and autonomic function below the level of their injury [1]. The global incidence of SCI is between 10 and 80 new cases per million people each year [2], with 4400 new cases per year in the United Kingdom [3]. The lifetime costs of SCI are estimated to total £1.43 billion (US $1.97) for 1270 cases per year in the United Kingdom [4].

The level of SCI is a major factor determining the degree of independence a person will achieve following injury [5]. Typically, the higher the level of injury, the lower the level of independence [6]. Damage to the cervical spinal cord results in tetraplegia, where function of all 4 limbs is impaired [7]. As well as physical and sensory issues, people with SCI are at risk of pressure ulcers, urinary tract infections, spasticity, autonomic dysreflexia, depression, neuropathic pain, difficulty breathing, and circulatory problems [8]. This multitude of impairments following SCI is associated with lower quality of life [9].

Immediately following injury, patients with SCI require acute medical management. This is followed by a period of rehabilitation as an inpatient. The aim of rehabilitation post SCI is to optimize recovery, restore and maximize function by leveraging activity-dependent plasticity for neurological recovery, learn compensatory techniques for lost function, and prevent secondary complications [10]. Central nervous system plasticity, where neural pathways can be altered in response to an injury and promote recovery, can occur spontaneously and/or as a result of rehabilitation [11]. In the last 30 years, the primary aim of physical rehabilitation of SCIs has moved away from compensatory strategies to neurological recovery, particularly in people with incomplete SCI [12]. Incomplete SCI refers to the preservation of sensory and/or motor function below the level of injury, as opposed to complete injuries, which do not have preservation of sensation or movement [7]. Rehabilitation is lifelong, but it is recommended that rehabilitation starts early after injury to maximize functional status and clinical outcomes [10].

Reducing reliance on care and achieving higher levels of independence with respect to activities of daily living is a major goal for patients and therapists [13]. Improving motor function, especially of the upper limbs, through rehabilitation [14] enables patients to carry out tasks, such as dressing, bladder and bowel care, transferring in and out of a wheelchair, and feeding which may otherwise require a carer [15-17]. Even small improvements in upper limb function can have large effects on a patient’s independence [18,19]. It is understandable, therefore, that improving upper limb function is a high priority for people with tetraplegia [13,20-22].

Improvements in upper limb function can be achieved through Activity-Based Therapy (ABT) [23]. ABT, a rehabilitation approach, refers to any intervention that involves high-intensity, repetitive exercises that target activity-dependent plasticity in spinal circuits [24]. The improvements in upper limb function from ABT have greater effects on quality of life when compared to traditional physical interventions targeted above the level of injury [19].

Virtual reality (VR) technology has the potential to deliver upper limb therapy by providing engaging interventions for patients as part of their rehabilitation [25,26]. Immersive VR delivers to the user a real-time digital environment that incorporates multimodal sensory paradigms, including proprioception, kinesthesia, stereopsis (the perception of depth via a stereoscopic display), and spatial audio, along with the ability to interact within the digital environment via head and hand tracking [27]. In these environments repetitive upper limb movements, like ABT, can be performed. VR-based therapy can facilitate greater adherence to therapy [28] and increase access to the most effective rehabilitation strategies for people with neurological disorders [27]. However, currently there are few studies that have investigated the use of VR in rehabilitation of the upper limbs, with most focusing on the chronic stage after injury.

VR-based upper limb therapy has the potential to provide engaging interactive environments within which patients can undertake the same type of movement repetitions as they would in conventional therapy. Due to its immersive qualities, patients may be more motivated to engage in therapy and may be able to complete more repetitions than they would compared to conventional treatment. A total of 6 reviews published between 2019 and 2024 have evaluated the evidence for VR for upper limb rehabilitation following SCI. These reviews included between 3 and 7 studies involving the upper limb. The overall findings from these reviews suggested that the evidence base is limited and that immersive VR appears to be more beneficial than non- or low-immersive VR [28-31]. However, these studies also suggested that exercises delivered in immersive VR could be used as an adjunct to conventional rehabilitation, which requires further investigation.

The VR devices, software, and methodology surrounding implementation used for upper limb therapy vary widely. VR-based interventions also vary in terms of their intended clinical use, fidelity, and cost, and the design and production of the software have rarely incorporated the lived experiences of SCI experts (people with SCI and health care professionals). The input of end users into the development of software, particularly in health care, is important in delivering successful products [32-34]. This process allows highly customized and bespoke projects to be developed, ensuring that the service or intervention is targeted toward the needs of patients [33].

This study forms the first part of a cocreation process for the design of a suite of VR-based exercises for upper limb rehabilitation in the acute SCI rehabilitation context. The aim of this study was to explore the lived experiences of people with tetraplegia and specialist SCI therapists related to acute upper limb rehabilitation and to co-design immersive VR-based upper limb activities.

## Methods

### Overview

In this qualitative study, 7 focus groups were conducted online from August to October 2022 to gather opinions and insights from the lived experiences of people with tetraplegia and, separately, SCI specialist therapists with experience of upper limb rehabilitation.

### Recruitment (Procedure)

A convenience sample of people with (1) tetraplegia and (2) therapists specializing in SCI rehabilitation were recruited. Although the VR intervention was to be developed for inpatient SCI rehabilitation, in order to fully explore lived experiences of participants with tetraplegia, the study recruited people who had been discharged from hospital having completed rehabilitation.

Participants with tetraplegia were recruited through the third sector organization Spinal Injuries Scotland. Recruitment material was also shared on social media channels (Twitter and LinkedIn). Inclusion criteria for participants with tetraplegia were a diagnosis of nonprogressive tetraplegia, aged ≥18 years, able to attend focus groups and contribute in spoken English, and having been discharged from hospital after receiving upper limb rehabilitation in a spinal injuries unit as part of their treatment. Exclusion criteria were any comorbidities that could preclude participants from attending and contributing to focus groups.

Therapists (occupational therapists and physiotherapists) were recruited through similar social media channels and a WhatsApp group of United Kingdom and Ireland–based SCI rehabilitation specialists. Inclusion criteria for therapists were being aged 18 years or older and able to attend and contribute to focus groups in spoken English.

Respondents to recruitment material emailed AG to register interest in participating in the study. Participant information sheets were provided to these interested parties by email. Participants provided informed consent prior to taking part in the study by either printing and physically signing a digital consent form or completing the form digitally. Participants without the physical ability to sign consent forms provided consent by designating a witness to sign on their behalf [35].

After providing informed consent, participants were asked to complete a demographic information form; for people with tetraplegia, this included their age, sex, Spinal Cord Independence Measure self-reported score [36], and for therapists, this included sex, occupation, and years of professional experience.

### Focus Groups

Focus groups were chosen over individual interviews to promote discussion and ideation between focus group participants. Question schedules were created to enable semistructured discussion between participants (Multimedia Appendices 1 and 2). The questions were devised to enable future software design and software requirements specification. The difference between the question schedules and conduct of each focus group was minimal. Participants with tetraplegia focus groups aimed to explore the topic of VR through the lived experience of participants with tetraplegia, and therapist focus groups aimed to explore the topic through the lens of clinical practice. Each focus group had 2 facilitators, AG (male) and LP (female). A total of 3 focus groups of therapist participants and 4 focus groups of participants with tetraplegia were conducted. The duration of focus groups ranged between 45 and 75 minutes.

Participants with tetraplegia were asked to recall their experiences of the acute stage of inpatient SCI rehabilitation. They were then asked to discuss upper limb impairments caused by SCI and how these relate to prehension, strength, and range of motion. Therapists were instead asked about the current practice surrounding delivery of upper limb therapy. Both groups were introduced to the concept of VR as a commercial product for immersive entertainment. They were shown a video promoting a high-immersion head-mounted display being used by able-bodied people to play interactive games [37]. Both cohorts were then asked to discuss their opinions on VR as an adjunct to usual upper limb rehabilitation and the potential facilitators and barriers to its use in the acute rehabilitation setting. Finally, specifications for the design of a VR system to promote upper limb exercise were gathered from both groups.

### Analysis of Transcripts

Focus groups were audio-recorded, and recordings were transcribed verbatim following pseudonymization of participants. Transcriptions were generated manually to enable familiarization with the data. Content analysis was used to count the responses and determine the meaning of participants’ discussions via code generation [38]. Therapist and tetraplegia focus groups were analyzed separately. After codes were generated by AG, they were independently reviewed by LP, M Poyade, and M Purcell*.* AG, LP, M Poyade, and M Purcell discussed codes to come to a consensus on identification of codes and naming of themes in the theme hierarchy. Quotes are presented with participant identifiers and their focus group number. Data were managed using NVivo (version 20; Lumivero) [39].

Codes (units of meaning) were typically sentences, phrases, or longer portions of text. Shorter phrases and sometimes single words were coded if they related specifically to recommendations for the design of the VR games. Initially, codes were organized under categories deduced from the schedule of questions. Subthemes were abstractions of groups of similar codes and were created during coding. Major themes were then generated from groups of subthemes and replaced the organizational categories. Transcripts were not returned to participants for comments or corrections.

### Reflexivity

Relationships with study participants were not established prior to study commencement. LP had previous experience facilitating focus groups and interviewing research participants, but AG did not. Neither facilitator worked in SCI rehabilitation and, as such, reduced the likelihood of inhibiting frank discussion about patient care with participants [40].

## Results

### Participants (Demographics)

A total of 18 participants with tetraplegia and 14 therapists expressed interest in the study. Of these, 3 therapists and 3 participants with tetraplegia did not attend the focus groups; thus, 15 people with tetraplegia and 11 therapists took part (Table 1). We conducted 3 focus groups with therapists and 4 focus groups with participants with tetraplegia. A total of 7 participants with tetraplegia did not complete the demographic information forms. Reasons for not completing the information form included technical difficulty completing the form (reported only by participants with tetraplegia). A total of 3 therapists did not report their years of professional experience. Therapists reported not having enough time or opted out of form completion, with no reason given. Half of the participants with tetraplegia had experienced immersive VR at least once, but none had used VR extensively.

**Table 1:** Summary demographic information for participants with tetraplegia and therapist participants.

Characteristic				Value
**Participants with tetraplegia (n=15)**				
	SCIM^a^ score^b^ (n=8), mean (SD)			17.9 (6.4)
	Age (years; n=8), mean (SD)			54.5 (8.6)
	**Sex (n=13), n**			
		Male	10	
		Female	5	
	**Experience with VR^c^ (n=8), n**			
		None	4	
		Some	4	
**Therapist participants (n=11)**				
	**Professional background (n=11), n**			
		Occupational therapists	7	
		Physiotherapists	4	
	Mean years of professional experience^d^ (n=8)			14.8 (SD 8.6)
	**Sex (n=11), n**			
		Male	2	
		Female	9	

^a^SCIM: Spinal Cord Independence Measure.

^b^Only 8 out of 15 participants with tetraplegia completed the SCIM and age information; 13 participants reported sex. Lower SCIM scores indicate lower independence (total score range, 0-75). Only 15 out of 19 subscales were included, comprising 6 self-care items and 9 mobility items.

^c^VR: virtual reality.

^d^A total of 3 therapists did not report their years of professional experience.

### Major Themes and Subthemes

#### Overview

**Figure 1:**
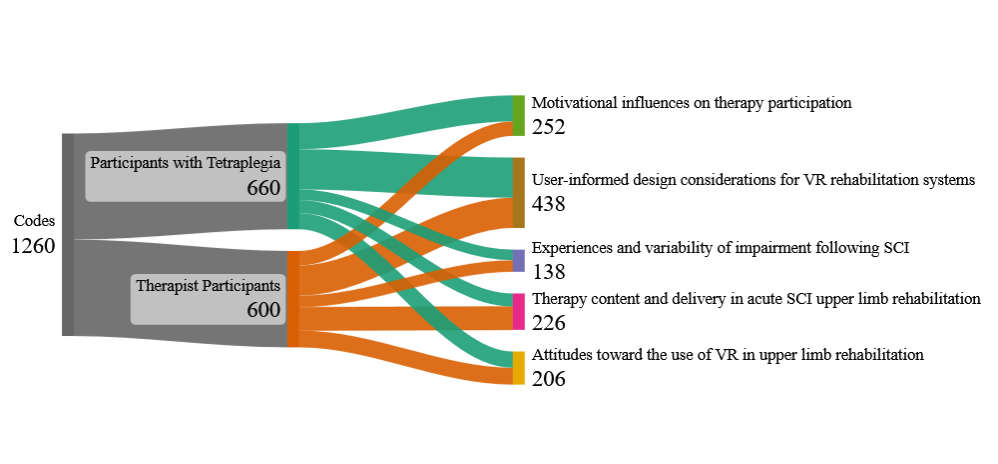
Visual summary of the number of major themes (listed in the right column) identified from the 1260 codes (shown in the left column) generated by content analysis of focus groups with participants with tetraplegia and therapists. SCI: spinal cord injury; VR: virtual reality.

Analysis of the focus group transcripts identified 5 major themes shared by the therapists and participants with tetraplegia: descriptions of impairment, descriptions of acute rehabilitation, factors affecting participation in therapy, perspectives on VR, and suggestions for VR design. A total of 35 subthemes were generated from 660 and 600 individual codes from the tetraplegia and therapist groups transcripts, respectively (Figure 1).

#### Major Theme 1: Experiences and Variability of Impairment Following Spinal Cord Injury

A total of 5 subthemes were identified that described impairments resulting from SCI immediately after injury and during the acute stage of rehabilitation (Table 2). Participants described profound loss of upper limb movement following injury, with variable patterns of recovery.

**Table 2:** Summary of subthemes for the major theme “experiences and variability of impairment following spinal cord injury.”

Subtheme	Therapist codes, n	Participant codes, n	Total, n
Impairment of upper limb movement	17	28	45
Other impairments	27	8	35
Impairment of function, including activities of daily living	9	19	28
Heterogeneous presentations and demographics	16	2	18
Impairment of upper limb strength	3	9	12

My arms, I wasn't able to move them at all after I had my accident.

PwT27, FG 3

Impairments were described as highly variable across individuals and influenced by the level and severity of SCI.

I suppose [the degree of upper limb impairment] depends on the severity of your tetraplegic-ness, you know. Some people might, might be really bad and some people might still have a bit of movement.

PwT27, FG 3

Both groups, but especially therapists, discussed “other impairments.” These included spasms, pain, and mental health issues, with therapists also raising age-related comorbidities and complications.

Therapists specifically raised the concept of “heterogeneous presentations and demographics” in acute SCI, with a growing prevalence of central cord syndrome in older adults, presenting as greater weakness in the upper limbs (than lower limbs) and greater involvement of distal muscles [7].

You could have somebody who’s got, you know, full shoulder movement, full elbow movement, but they’ve got some weakness in the hands. Or the opposite, you know you could have someone who has got good hands but reduced movement at the shoulder.

TP3, FG 1

#### Major Theme 2: Therapy Content and Delivery in Acute Spinal Cord Injury Upper Limb Rehabilitation

A total of 7 subthemes described the nature of inpatient rehabilitation following SCI (Table 3). Upper limb therapy was described as a combination of functional and remedial activities, with a strong focus on maintaining range of movement and developing task-specific skills.

**Table 3:** Summary of subthemes for the major theme “therapy content and delivery in acute spinal cord injury upper limb rehabilitation.”

Subtheme	Therapist codes, n	Participant codes, n	Total, n
Upper limb therapy	92	51	143
Patient-centered care	31	0	31
Assistive devices, orthotics, and neck stabilization	0	16	16
Barriers to effective therapy	12	0	12
Psychological adjustment and treatment	4	9	13
Bed rest and ventilation	0	5	5
Symptom management	6	0	6

[…] we do work on range of movement, you know shoulder, elbow, wrist, fingers.

TP7, FG 2

There were various things that we used—we had these games where we had little pegs that we had to move around the board, or also another one with a bucket full of the marbles.

PwT16, FG 4

Therapists also discussed upper limb functional improvement, particularly where patients have incomplete injuries:

If they’re an incomplete injury we might be looking for more of a normal movement. […] Whereas if they’re more of a complete, it’s obviously looking at function and how we can use what they’ve got—what muscles they’ve got working to functionally achieve the task.

TP9, FG 2

Both groups highlighted the common use of “assistive devices” during rehabilitation, which included arm and hand stabilization using orthotics and neck stabilization, which hindered movement.

I had to use the Active Hand, which are the Velcro gloves so I could hold things.

PwT16, FG 4

I was 12 weeks in a neck brace, so I ended up with a very, very weak neck.

PwT13, FG 3

Under therapist-only subthemes, patient-centered care was achieved through collaborative goal setting. Barriers to therapy delivery included delayed referral and limited resources. Psychological adjustment was described by both groups, with participants with tetraplegia emphasizing loss of independence and therapists focusing on expectation management.

We would set those goals collaboratively with the patient based on a little bit of, kind of advice and guidance from us as to what we anticipate their outcomes might be.

TP5, FG 1

Unfortunately, the later we get someone sometimes the more issues we can have with contractures and upper limb pain and things, which then makes it harder for us then to, to rehab them.

TP9, FG 2

Trying to deal with everything and I found it really hard to be having to rely on other people for your personal care just day in, day out for everything.

PwT21, FG4

In their mind they want [a goal] to be achievable. So it, again comes down to communication and managing those expectations of patients and their families.

TH9, FG2

“Bed rest and ventilation” and “symptom management” were less commonly raised in both groups.

#### Major Theme 3: Motivational Influences on Therapy Participation

A total of 10 subthemes described factors influencing participation in therapy. Subthemes were then classified as “motivational” or “demotivational” (Table 4). Motivational influences included task enjoyment, goal setting, peer interaction, and visible improvement. Participants with tetraplegia described greater engagement with game-like and functional activities compared to repetitive remedial exercises.

**Table 4:** Summary of subthemes for the major theme “motivational influences on therapy participation.” Subthemes are categorized as either promoting motivation or reducing motivation.

Subtheme	Motivational or demotivational	Therapist codes, n	Participant codes, n	Total, n
Useful or enjoyable rehabilitation	Motivational	1	39	40
Goal setting and therapist support	Motivational	6	31	37
Understanding tasks and prognosis	Motivational	30	0	30
Peer support and competition	Motivational	5	11	16
Seeing improvement	Motivational	0	10	10
Boring, repetitive, or difficult rehabilitation	Demotivational	5	41	46
Limiting symptoms	Demotivational	22	6	28
Low mood	Demotivational	11	6	17
Downtime	Demotivational	0	15	15
Limited resources and lack of supervision	Demotivational	10	3	13

We had some games to play, which is quite good fun. Like maybe like playing dominoes. Uh, so fiddling about with them things and also trying to build things with Lego was quite motivational and we had a tower of blocks that was probably my favourite thing.

PwT26, FG 4

In contrast, “boring, repetitive, or difficult rehabilitation,” which included pointless, tedious, and humiliating activities, was demotivational.

You know what it’s like sometimes you go and do a bit of exercise, you know you’re pulling on a TheraBand and [imitates snoring] ‘boring!’.

PwT1, FG 1

Sometimes strategies that were intended to make therapy more enjoyable, such as creative activities using putty or interactive cycling, were frustrating for patients.

[…] most days doing the exercises with the putty and so on, it felt like a really kind of childish, I don't know. Like, ‘what's this doing?’

PwT21, FG 4

And [patients] found that really—you know they were cycling [FES cycling] away, cycling away along this road but they never went anywhere. And they got really frustrated by that.

TP9, FG 2

Some activities were too difficult or too easy for participants with tetraplegia, which resulted in them disengaging from therapy.

I also had a board with different sizes of bolts and nuts that I had to screw and I hated that because I thought, you know, ‘I can't do this! Why are they making me do this when I cannot do it? This is so humiliating’.

PwT16, FG 4

Therapist discussions described patients were more motivated if they understood their injury, prognosis, and purpose of therapy (“understanding tasks and prognosis”).

[Patients need] to have a good understanding of their injury and what the task is working towards. If they don’t then often they’re: “What’s the point?” or “I don’t really know why I’m just rolling this putty out”. Umm “I’m not gonna get any better” or “How’s this gonna strengthen?”. So how much they understand about their injury is really important in their engagement.

TP5, FG 1

Both groups also raised that “peer support and competition” could be motivational.

Some of our patients…. a kind of element of challenge or competition almost with themselves in some of the more repetitive remedial tasks, um, can be really beneficial—like if they get 10 pegs one day - the next time they want them to get 15.

TP4, FG 1

I think being in the group as well gave you a bit more, you know, just a bit more reason to sort of get on with it and people would be chatting and you had that, you know, nice social aspect as well.

PwT27, FG3

Demotivational factors included repetitive or infantilizing tasks, symptom burden, low mood, and comparisons with faster-progressing peers.

Other people were whizzing through, and you just sat there doing like one at a time. And it was just demoralizing.

PwT21 FG 4

[…] a lot of the time I was just knackered, it turned out that I had an underlying infection due to the metalwork in the back of my neck.

PwT1, FG1

A lot of pain can be difficult for them to kind of adhere to upper limb therapy (or any therapy).

TP4, FG 1

I mean, some people in the unit—when I was there for 12 months didn't do anything. And they just lay in bed. They gave up.

PwT4, FG 2

Disengagement could occur if therapists could not always be present during therapy (“limited resources and lack of supervision”).

[…] you know people who never do what’s asked of them unless someone’s standing over them making them do it.

TP12, FG 3

You know, they did their best to keep things fresh and, and try introducing, but obviously they had a limited amount of resources available to them, but equally limited staff time.

PwT13, FG 3

Finally, participants with tetraplegia identified “downtime,” especially at the weekend, which meant there was nothing to do, and, as such, was demotivational.

The weekends at the spinal unit are, are very long and you just you know everything finishes on a Friday and you do nothing till Monday and you just feel I could I could be doing [something].

PwT27, FG 3

#### Major Theme 4: Attitudes Toward the Use of VR in Upper Limb Rehabilitation

A total of 8 subthemes captured attitudes toward VR (Table 5). Participants described VR as a potentially engaging and meaningful adjunct to therapy, particularly for individuals who struggle to engage with conventional rehabilitation.

**Table 5:** Summary of subthemes for the major theme “attitudes toward the use of virtual reality in upper limb rehabilitation.” Subthemes are categorized as having a positive or negative sentiment.

Subtheme	Positive or negative sentiment	Therapist codes, n	Participant codes, n	Total, n
VR^a^ could/should be engaging and meaningful	Positive	14	31	45
VR could/should be used for rehabilitation	Positive	9	18	27
Younger patients will like VR	Positive	9	0	9
VR could alleviate symptoms	Positive	5	4	9
Consumer equipment is not suitable	Negative	29	15	44
VR could cause adverse effects/be inappropriate	Negative	17	33	50
Resources are limited	Negative	15	0	15
Training is required	Negative	7	0	7

^a^VR: virtual reality.

So I think VR would have a very big use in a spinal injury centre there’s a whole range of things it could cover. It’s exciting it would be fun to use with people.

TP8, FG 3

I think [VR] is great because as I said earlier, there are other people [in hospital] who, you know, their mindset is totally negative. […] they need that stimulation, that encouragement, and VR might be the way.

PwT4, FG 2

[VR could] make any training or rehab more, more interesting, more exciting, more varied. Yes, I think… huge potential.

PwT13, FG 3

Both groups said that VR could encourage engagement in therapy by reducing symptoms such as pain and improve mood (“VR could alleviate symptoms”).

And [VR is] good, actually help people to get involved in activities without even imagining the pain they are in.

PwT19, FG 3

I do quite like that element of escapism for them, I think. It could psychologically really help them as well.

TP3 FG 3

Conversely, concerns were raised from both groups regarding equipment suitability, adverse effects, staffing capacity, and training requirements. Head-mounted displays were described as potentially heavy and unsafe for individuals with high-level injuries.

If [devices] are too labour intensive or take a while to set up or need someone there then when we’re short-staffed as we have unfortunately been for quite a long time, um, you tend to go for things that are quicker and easier to use if you’ve only got quite a short treatment session.

TH9, FG2

The other thing as well is our very high injury levels, [who are] struggling just holding their head up—or that kind of movement with the head [TP8 demonstrates neck rotation] a lot—it can tire them.

TP8, FG 3

Both groups warned that “VR could cause adverse effects” such as nausea, or that the specific activities in VR that could remind them of their SCI.

I think when you’re already feeling self-conscious about yourself as an individual and how you move… VR might have a bit of a negative effect on people as well.

TP5, FG 1

When you take [the VR headset] off and you go ‘Oh shit. It’s true, this is where I am’.

PwT21, FG 4

Therapists speculated that their younger patients would be more suited to VR games (“younger patients will like VR”).

So I think the younger people will probably get [VR], you know they’ll understand a lot better how it works.

TP4 FG 1

#### Major Theme 5: User-Informed Design Considerations for VR Rehabilitation Systems

A total of 5 subthemes described participant recommendations for VR system design (Table 6). Suggested activities ranged from functional or vocational tasks, suggested mostly by therapists, to leisure, sporting, and creative environments, which were suggested predominantly by participants with tetraplegia. Preferences varied widely, reflecting differences in interests and functional abilities.

**Table 6:** Summary of subthemes for the major theme “user-informed design considerations for virtual reality rehabilitation systems.”

Subtheme	Therapist codes, n	Participant codes, n	Total, n
Activities and scenarios	86	150	236
Usability and training	32	33	65
Measuring performance and achievement	38	27	65
Movements	21	23	44
Immersion, presence, and engagement	11	17	28

[…] it's down to the individual what interests they have and you know what would benefit them the most and… I mean, metalwork is obviously not gonna be the same as scuba diving and, you know, skiing, or playing the piano. One’s having loads of fun, one’s more sort of creative and you're constructing something in VR. Yeah. So yeah, just depends.

PwT4, FG 2

Therapists also discussed providing a range of different activities for their patients. Both groups recommended incorporating concepts such as relaxation and escapism into the design (“immersion, presence, and engagement”).

So we are really trying to provide a variety of different type of games that we offer but obviously when you’ve got a patient who’s had a long (and all of our spinal injury patients have quite a long) admission with us, it’s like the variety that’s key.

TP14 FG 3

What about making really realistic sound, […] soaring noises, painting noises of the paint going on to the wall and those kind of things where it actually feeds back extra reality to the experience?

PwT16, FG 4

Additionally, participants with tetraplegia and therapists suggested outdoor spaces such as hills, glens, mountains, underwater spaces, outer space, and jungles would be good environments for VR activities.

[I have] a static bicycle at home. I get a bit bored because I’m always in the same place, while if you could have the feeling that you're actually outside and going places that might make it more interesting.

PwT16, FG 4

Both groups said that VR games should enable a range of movements, from fine to gross (“movements”), including reaching, grasping, and wrist flexion and extension.

[VR] could be something that could be really useful for larger gross movements but also for really drilling down to repetition of very fine motor movements.

TP12, FG 3

Therapists also recommended the quality of movements in VR was of high importance.

Sometimes it’s not the number of movements, it’s quality as well. So, you know, we’d be looking at the quality of someone’s reach, not just that they are able to reach, but maybe not recruiting the right muscle groups to perform that task.

TP9, FG 2

Both groups gave recommendations for “measuring performance and achievement” of users. These included giving encouragement and providing feedback to increase engagement. Therapists discussed providing incremental levels of difficulty to accommodate progression and achievement.

[…] like you get a big tick or a big thumbs up or something. Not something annoying but something that shows you've reached the level of the activity that's actually doing something.

PwT27, FG 4

Therapists recommended incorporating easily comprehensible progress reports and feedback to users to help with motivation.

[…] and patients love seeing kind of that—we can put it—display it on a graph for them to kind of say like this is what you did like a month ago and this is what you’re doing now.

TP8, FG 3

Therapists and participants with tetraplegia recommended that the movements should be configurable for users, and that training should be provided to patients and therapists (“usability and training”).

So there’s gonna have to be sort of that adjustability with the system to make it relevant to that patient’s rehab and what muscles you want to work on and that, yeah.

TP7, FG 2

Furthermore, under this subtheme, it was highlighted that adding straps to the controllers might be required, and for those with limited hand function, participants with tetraplegia also requested interaction mechanisms using head-movement, gaze, hand tracking, and voice input.

I think you'd have to be really aware of even things like how people could grip any hand controls, you know, just the simple things like holding them, putting headsets on, how much assistance was needed to make it as practical as possible.

PwT21, FG 4

### Ethical Considerations

Ethical approval for this study was granted by the Psychology, Social Work, and Allied Health Sciences (PSWAHS) Research Ethics Committee of Glasgow Caledonian University (reference: HLS/PSWAHS/21/247). Participants with tetraplegia were recruited via a gatekeeper (Spinal Injuries Scotland). Therapist participants were recruited via social media. Both groups were provided with participant information sheets that detailed the aims of the study and, if they chose to take part, the requirements of participation and the information collected about them. This information would be stored on Glasgow Caledonian University’s OneDrive for 5 years and shared only with the study team. All information would be anonymized after consent was given, and participants assigned an ID number to comply with the General Data Protection Regulation (GDPR). Prior to participation, all participants provided informed consent either by printing and physically signing a digital consent form or by completing the form digitally. Participants who were unable to physically sign the consent form provided consent through a designated witness who signed on their behalf [35]. The study abided by the guidelines set out in the Declaration of Helsinki. Participants were not compensated for their participation and were informed they were free to withdraw from the study at any time without giving a reason. Participants could have their information removed from the study but were informed that this would not be possible after anonymization.

## Discussion

### Principal Results

The aim of this qualitative study was to explore the lived experiences of people with tetraplegia and SCI specialist therapists in terms of upper limb rehabilitation to inform the design of a VR-based intervention. A total of 5 major themes and their associated subthemes were identified that enabled the specification of software requirements, which have subsequently been implemented in a prototype VR intervention. The focus groups indicated that VR has the potential to help improve function and build strength, as well as learning skills, by adapting to loss of function. This suggests that end users of potential VR-based interventions understand its possible benefits, as well as pitfalls. The results also show that end users see a place for co-designed interventions in the acute/subacute setting and showed interest in VR, which is critical for “buy-in” for the adoption of new technologies.

### Major Themes 1 and 2

The findings indicate that VR-based interventions must accommodate substantial variability in upper limb impairment following SCI. To account for diverse user profiles and needs, software must adapt to the personal rehabilitation needs of the user. Although some therapists anticipated uptake among younger patients, and therefore specific targeting toward this demographic, restricting designs to this group would misalign with the clinical diversity of the population. Design should therefore prioritize broad accessibility and usability across impairment, age groups, and levels of technological expertise.

### Major Theme 3

This study highlights how motivation and engagement are shaped by both individual and service-level factors during acute/subacute rehabilitation. The findings align with established barriers to engagement in therapy in the SCI literature, including comorbidities (eg, urinary tract infections, pressure sores, fatigue, pain, and low mood), alongside the resource limitations of health care providers, periods of “downtime,” variation in individual rehabilitation needs, and patients’ desire for more control over their therapy [41-44]. Patients with SCI were generally described by both groups as highly motivated, with engagement closely linked to perceived progress and the pursuit of greater independence. This finding may be influenced by sampling bias, as individuals willing to participate in research may be more motivated than the broader inpatient population. Motivational drivers suggest that interventions incorporating visible progress indicators and structured challenges may be particularly effective. Such features could also increase acceptability of novel interventions, including VR therapy, when used as an adjunct to conventional rehabilitation.

### Major Theme 4

Participants with tetraplegia described VR as an assistive technology to enhance function and therefore improve independence, a factor shown to be of high importance to people with SCI [45]. In addition, VR was perceived as a tool to give participants with tetraplegia independence during their rehabilitation. Participants’ emphasis on autonomy and meaningful activity suggests that VR may be most effective when it enables users to exercise choice and engage in personally relevant tasks. These factors could also enable therapists in administering or prescribing therapy by facilitating movements that are typically difficult to achieve in usual treatment or that are more appealing to people with tetraplegia.

The repetitive nature of upper limb rehabilitation presents an opportunity for VR to embed prescribed movements within more engaging tasks. VR could maintain motivation by using repetitive input to drive engaging game mechanics, such as firing a bow and arrow, hitting boxing targets, or completing puzzles. Progress-based feedback and structured challenges may further promote sustained participation.

Users could be engaged in gameplay and rewarded in immersive scenarios for repeating movements. This concept relies on the immersive capabilities of VR to provide positive distraction, which is a term inferred from participants’ discussion regarding “escapism,” from typical therapy while still enabling prescribed movement.

The acute context of rehabilitation is relevant, as patients must adapt to both sudden functional loss and prolonged hospitalization. Participants highlighted the importance of experiences that offer psychological relief from this environment via relaxation and escapism, which are achievable with immersive VR systems [46].

Resource limitations were identified as a barrier to therapy delivery by both groups. Participants implied that a VR-based system could mitigate these limitations by enabling self-guided, semi-independent practice. This could allow therapists to allocate time more efficiently while maintaining a supervisory role in therapy sessions.

The group-based nature of rehabilitation suggests that future VR systems could benefit from supporting multiuser networked environments to encourage peer-to-peer interactions.

### Major Theme 5

Both therapists and participants with tetraplegia made many suggestions about what type of activities and upper limb movements they would like to see implemented in VR. There was no consensus on particular types of VR scenarios with which patients with tetraplegia would engage, reflecting the heterogeneity of the tetraplegic population. This supports the development of flexible systems capable of offering varied activities and environments to their users.

VR is most likely to add value when repetitive therapeutic movements are embedded within adaptive, gamified tasks. These findings are consistent with existing research on rehabilitation during acute/subacute tetraplegia [45,47-50]. Participants’ emphasis on independence, motivation, and shared decision-making reinforces the important of human-centered design principles. Unique to this population is the complexity of SCI, which remains a central factor for consideration during intervention development.

These findings add to the growing body of research concerning the recommendations for implementing VR for people with upper limb impairments and their rehabilitation [51-54]. VR interventions must be designed to take advantage of their attributes, which include real-time feedback and immersive environments. These designs must be informed by the specific populations who are intended to use these systems [55-58].

### Recommendations for the Design of a VR Upper Limb Rehabilitation Intervention for People With Tetraplegia

The following recommendations outline key design considerations for VR-based upper limb rehabilitation interventions targeting people with tetraplegia. They are structured around practical software development domains to support systematic intervention design and informed by the themes identified in the focus group data.

#### Domain 1: Accessibility

VR applications should be usable by the widest possible range of people with tetraplegia and accommodate the wide variation in upper limb function of users for control of the system. They should be usable to the same extent for all users, irrespective of upper limb function, and support patient autonomy while maintaining the therapeutic role of clinicians. VR applications should be usable across age groups and comorbidities associated with tetraplegia, allow all prescribed movements to be specified and configured by the user, and must be comfortable to use. They should target as many movement domains as possible and support movement or activity that is difficult to achieve in standard upper limb treatment.

#### Domain 2: Implementation

VR applications should be feasible, fit into existing clinical workflows, and aim to alleviate resource limitations. They should support collaborative goal setting between patients and therapists and minimize physical and psychological adverse effects associated with, and attributable to, VR. Applications should consider the appropriate intensity and duration of an intervention and prevent overexertion during use. They should also be able to monitor usage and performance and be supported by appropriate training for end users to ensure safe implementation and effective integration into clinical services.

#### Domain 3: Immersion and Engagement

VR applications should increase and maintain engagement beyond what is achieved in usual treatment by encouraging repetitions of prescribed movements through meaningful virtual activities and environments. They should avoid factors that cause disengagement from therapy by providing an optimal degree of challenge for each user, ensuring that the experience is neither frustrating nor too difficult or too easy. Applications should provide motivational feedback on performance, such as scores and progress, and make use of the immersive and enjoyable qualities of VR technology to ensure users feel present within virtual environments and benefit from escapism or positive distraction.

### Limitations

Participants with tetraplegia sometimes had difficulty recalling their time as inpatients, typically when years or decades had passed following discharge. However, it was important to prioritize the perspective of individuals with a full retrospective view of inpatient care.

Separate focus groups were conducted for therapists and participants with tetraplegia to remove the effect of the relationships or perceived power differences between patients and clinicians. For example, when discussing opinions about particular types of upper limb therapy, participants with tetraplegia may have been reluctant to express negative opinions about care if therapists were present. Interaction between participants with tetraplegia and therapists may, however, have been beneficial in terms of ideating suggestions for the design of VR scenarios.

Data saturation was not achieved as the final focus groups in both groups yielded additional insights, though at a lower frequency compared to earlier groups. However, the information power of the focus groups was improved by revising the question schedules after each focus group. In particular, follow-up questions and prompts were added to explore new areas of discussion that had not been covered by previous groups. The inclusion of different rehabilitation disciplines with experience of working in different specialist centers across the United Kingdom ensured diverse groups were assembled, which was a strength of this study and allowed for more varied discussion.

### Conclusions

This study reported the perspectives and suggestions of people with tetraplegia and SCI therapists about the use of VR for upper limb rehabilitation in the acute setting. As a result, it presents valuable first-hand information about the design of VR technology for SCI rehabilitation. A total of 5 themes were generated that designers and developers of immersive applications for rehabilitation can use to create their applications. People with tetraplegia have a large variation in upper limb impairments, for which many different movements are prescribed by therapists. This variation must be accommodated by applications in a usable and accessible way. Applications must also implement features that motivate patients to attend and adhere to therapy, such as providing useful feedback and appealing to the broad range of patients’ interests, and also avoid factors that can cause disengagement from therapy. The findings of this study facilitated the cocreation of a novel immersive VR intervention for upper limb rehabilitation for people with tetraplegia while they are inpatients.

### Future Work

The next part of this project is to use the results from this study to produce software requirements and a software architecture for a suite of VR games for upper limb therapy. This study enabled the development and deployment of the proposed software and its continued improvement.

## Appendix

Multimedia Appendix 1 Schedule of Questions (PwT).

Multimedia Appendix 2 Schedule of Questions (Therapists).

## Abbreviations

ABT: Activity-Based Therapy
GDPR: General Data Protection Regulation
PSWAHS: Psychology, Social Work, and Allied Health Sciences
SCI: spinal cord injury
VR: virtual reality
