# Supplementary material for: Design Recommendations for Virtual Reality–Based Upper Limb Exercises From People With Tetraplegia and Spinal Cord Injury Rehabilitation Specialists: Focus Group Study
Source: JMIR Rehabil Assist Technol. 2026 Feb 11;13:e66832. doi: 10.2196/66832 (PMC12936658; doi:10.2196/66832)
Supplement: Multimedia Appendix 1 [file rehab_v13i1e66832_app1.docx]

Schedule of Questions – Participants with Tetraplegia.

The Co-Design of a Virtual Reality-Based Upper Limb Rehabilitation Platform.

**Introduction:** Thank you very much for joining us. We’re very grateful for your time and we’re really pleased you want to take part in our research. The main aim of this focus group is to gather information about your experiences of rehabilitation after spinal cord injuries.

We want to focus on your arms and hands. We would like you to talk about the time following your spinal cord injuries, when you were hospitalised and began rehabilitation. We want to know about what kind of exercise you did to improve your arms, and your opinions about the therapy you received.

[In-Person Only] Hopefully you will have the chance to try virtual reality – we will also ask about what you think about VR and how you can see it being used in spinal units to help people with their arm and hand rehabilitation.

[Ask participants to introduce themselves]

The questions are split into sections. The session should last about an hour [In-Person: an hour and a half].

**[VERBAL CONSENT – check that all participants continue to give their consent.]**

I am going to start the audio recording soon. Are you happy to continue with the focus group?

[Ask participants if they have any questions before the focus group starts]

[Start the recording]

**Part 1 (5 minutes):** Experience of upper limb impairments following spinal cord injury.

**About:** The first set of questions relates to the impairments to your arms and hands following your injury.

Question 1: Thinking back to the time shortly after your injury, while you were a patient in your spinal unit, how were your arms affected by your injury?

Prompt: Please describe what you could or couldn’t do with your arms.

Question 2: Thinking specifically about your impairment of your arms, how did this affect the things that you wanted to do using your arms and hands?

Prompt: What did you want to improve about your arms? What was most important to you about the recovery of your arms?

**Part 2 (20 minutes):** Experiences of current/past upper limb rehabilitation interventions, including factors motivating activity.

**About:** This set of questions relates to therapy that you received to improve your arms and hands.

Question 3: What kind of arm rehabilitation did you receive following your injury?

Follow-up: What kind of exercise did you do? Can you describe these exercises?

Question 4: How often were you supposed to do these exercises? And how often did you *actually* do these exercises?

Question 5: What aspects of rehabilitation were engaging? If so, what made it engaging? Again, think about rehabilitation related to your arms.

Question 6: Was there any aspect of rehabilitation that did not work for you? Why did it not work?

Follow-up: If you stopped doing a particular exercise, why did you stop?

Question 7: Thinking about the times when you didn’t feel like participating in rehabilitation, can you think about ways that could have motivated you to keep going?

Follow-up: What was motivating you when you were actively participating in rehabilitation?

Question 8: Can you think of any other ways that rehabilitation and exercise could be made more engaging, so that people want to keep trying it?

Follow-up: Did you have any way to keep track of your performance?

[Break]

**Part 3 (15 minutes):** Views on VR as an assistive technology to deliver Activity-Based Therapy and the barriers and facilitators to using VR with Acute SCI patients.

**About:** If VR was part of inpatient rehabilitation, patients would wear a headset and choose from a suite of different activity-based games or experiences. What a patient may actually see or experience in VR is undecided, and we would like to hear what your thoughts are about the technology.

[IN-PERSON GROUP ONLY - Show participants a VR headset and give opportunities to have a brief experience using VR. While not in VR, participants can watch the short video below]

**[Explain that participants are about to see commercial products. They have been designed and built by teams of people over several years]**

**[Explain that the video features people standing up using VR. However, we intend to design the VR therapy specifically for people with spinal cord injuries who have limited use of their arms and hands. Sitting down experiences]**

[ONLINE GROUPS – Participants will watch a short (<5mins) video that shows some of the capabilities of modern commercial VR head-mounted displays]
Video URL: <https://www.youtube.com/watch?v=qYfNzhLXYGc&t=32s>

Question 9: What are your thoughts about video you watched?

Prompt: Describe what you saw in the video. How did that make you feel?

Question 10: Compared to your experience of rehabilitation, what kind of advantages can you imagine VR would have?

Question 11: Can you think about any limitations or drawbacks to using VR?

Prompt: Do you have any concerns about using VR? Can you describe them?

Question 12: If you were given the opportunity to try rehabilitation using VR after your injury, would you have tried it?

Prompt: Can you explain why or why not?

**Part 4 (15 minutes):** Ideation about preferences for exercises and activities which could be undertaken in VR.

**About:** This set of questions aims to find out what you think would make engaging, fun, and effective activities in VR. Remember that the VR games are intended to improve the arms and hands of people who have recently had a spinal cord injury.

Question 13: Do you play any non-VR games? Could these games be combined with some form of arm exercise in VR?

Prompt: This could be any game, like cards, boardgames, sports, or video games, crosswords, etc.

Question 14: People often use VR to do things they couldn’t try in real life – visit a rainforest, fly an aeroplane, go to the surface of the Moon. Would this appeal to someone on a spinal unit? Why?

Follow-up: What kind of scenarios do you think would be engaging and interesting in VR?

Prompt: Think back to when you were an inpatient after your injury – what kind of things would you have wanted to try in VR then?

Question 15: Sometimes therapy is administered in groups although the therapy an individual receives is specific to them. Can you see VR being used in group situations?

Question 16: Is it important to you to get information/feedback about your progress related to rehabilitation?

Follow-up: Would that be beneficial to you?

Follow-up: And how do you think you could see this implemented in a VR game?

Prompt: If your therapists gave you feedback during rehabilitation, how was it delivered? Can you think of any other ways you could get feedback on your progress?

Question 17: What kind of exercises/arm/hand movements do you think would work well for you in VR?

Follow-up: What exercises do you think would not work very well for you in VR?

Conclude the session with a summary of the discussions, going over parts **1, 2, 3, and 4** of the focus group.

**Part 5:** Any other questions or ideas that the group wants to share.

[Ask the group if they would like to talk about anything else. Maybe there’s a point that they would like to make about something that’s already been spoken about, or something else entirely].

We would like to thank you for taking part in today’s focus group. Your contributions will be very useful for the next stages of this study, where we are going to use your responses to develop a suite of VR scenarios for rehabilitation.

Soon we will be looking to form an advisory panel of people with spinal cord injuries to provide feedback on the design and development of the VR games. If you are interested in contributing to the project please get in touch via email.

[End the recording]

[end]
