# Supplementary material for: Design Recommendations for Virtual Reality–Based Upper Limb Exercises From People With Tetraplegia and Spinal Cord Injury Rehabilitation Specialists: Focus Group Study
Source: JMIR Rehabil Assist Technol. 2026 Feb 11;13:e66832. doi: 10.2196/66832 (PMC12936658; doi:10.2196/66832)
Supplement: Multimedia Appendix 2 [file rehab_v13i1e66832_app2.docx]

Schedule of Questions – Participants who are Occupational Therapists/Physiotherapists.

The Co-Design of a Virtual Reality-Based Upper Limb Rehabilitation Platform.

**Introduction:** Thank you very much for joining us. We’re very grateful for your time and we’re really pleased you want to take part in our research. The main aim of this focus group is to gather information about your knowledge and experiences of rehabilitation after spinal cord injuries. We want to use the information we collect from this session to build a virtual reality intervention for upper limb rehabilitation in people with spinal cord injuries.

We want to focus on upper limb rehabilitation in the acute stage of SCI, in which functional improvements can have large impacts on independence of service users. We want to know about what kind of rehabilitation you provide for your patients and your opinions about the care they receive. We will also ask about what you think about VR and how you can see it being used in spinal units to help people with their arm rehabilitation.

[Ask participants to introduce themselves]

The questions are split into sections. The session should last about an hour.

[VERBAL CONSENT – check that all participants continue to give their consent.]

I am going to start the audio recording soon. Are you happy to continue with the focus group?

[Ask participants if they have any questions before the focus group starts]

[Start the recording]

**Part 1 (10 minutes):** Experience of treating upper limb impairments following spinal cord injury.

**About:** The first set of questions relates to treatment of impairments to the arms and hands following spinal cord injuries.

Question 0: Can you outline the services you provide for you patients?

Follow-up: How soon after their injury do you receive patients?

Question 1: What upper limb impairments can a spinal injury cause?

Prompt: Every patient is different – how do patients present in your services in relation to upper limb impairments? ~~but are there common patterns of injury you see in patients?~~

~~Question 2: How do these impairments affect a patient’s daily life?~~

~~Question 3: What are the outcomes of your services for upper limb rehabilitation?~~ ~~What do you aim to improve (in the upper limbs) when a patient is admitted?~~

~~Prompt: For example, do you administer therapy that helps improve dexterity?~~

~~Prompt: What other domains of impairment do you target?~~

Question 4: What movements or actions do you ask your patients to do with their arms and hands?

Follow-up: What impairment domains are these movements targeting?

Prompt: For example, do you administer therapy that helps improve dexterity?

Prompt: What other domains of impairment do you target?

~~Question 4: What can patients do after activity-based therapy, that they may not be able to do immediately after injury?~~

**Part 2 (15 minutes):** Experiences of current/past upper limb rehabilitation pathways, including factors motivating activity.

**About:** This set of questions relates to therapy that patients receive to improve their arms and hands.

~~Question 5: Can you describe the therapy that patients receive (for their upper limbs after their spinal cord injuries)?~~

~~Follow-up: What impairment domains do these activities target (for example strength, dexterity etc.)?~~

~~Follow-up: What are the main aims of upper limb therapy in acute SCI rehab?~~

Question 6: What kind of difficulties do patients have with therapy?

Follow-up: How long do they do them for and do they adhere to them?

~~Question 7: Is it important that rehabilitation is engaging?~~

~~Follow-Up: What aspects of rehabilitation are engaging and why?~~

Question 8: Are there barriers to therapy for patients? What are they?

Follow-up: What can motivate patients with their therapy?

~~Question 9: What motivates patients to keep trying an exercise?~~

~~Question 10: What challenges do you (therapists) have when supporting upper limb rehabilitation in acute spinal cord injuries?~~

**Part 3 (15 minutes):** Views on VR as an assistive technology to deliver Activity-Based Therapy and the barriers and facilitators to using VR with Acute SCI patients.

**About:** If VR was used as part of a service user’s rehabilitation in the acute stage of their injury, patients would wear a headset and choose from a suite of different activity-based games or experiences. What a patient may actually see or experience (for this study) in VR is undecided, and we would like to hear what your thoughts are about the technology.

[ONLINE GROUPS – Participants will watch a short (<5mins) video that shows some of the capabilities of modern commercial VR head-mounted displays]

**[Explain that participants are about to see commercial products. They have been designed and built by teams of people over several years]**

**[Explain that the video features people standing up, however we intend to design the VR intervention specifically for people with spinal cord injuries who have limited use of their arms and hands – sitting down experiences]**

Video URL: <https://www.youtube.com/watch?v=qYfNzhLXYGc&t=32s> **only show about 2 mins of video** ++ Google Drive Link: <https://drive.google.com/file/d/16XYYmt2WpWmwP8aLHXlaT_D8zCO34YRy/view?usp=sharing>

Question 11: What are your thoughts about virtual reality as you have seen it?

Question 12: Can you see VR being used as a tool for rehabilitation? Why or why not?

~~Question 13: Can you think about any limitations or drawbacks to using VR?~~

~~Prompt: Do you have any concerns about using VR? Can you describe them?~~

**Part 4 (20 minutes):** Ideation about preferences for exercises, activities, and parameterisation of a VR intervention.

Note: do not say things like ‘parameterisation’ – stick to the **About** section below.

**About:** This set of questions aims to find out what you think would make engaging and effective activity-based VR games. Remember that the VR games are intended to improve the arms and hands of people who have recently had a spinal cord injury.

Question 15: What kind of physical activities/limb movements do you think would be suitable for your patients in VR ~~for a VR intervention~~?

Follow-up: Are there any exercises that are difficult to do conventionally that would be particularly suited to VR?

Question 16: What kind of scenarios do you think would be engaging and interesting for your patients?

Prompt: Try to imagine some virtual environments or scenarios that patients would engage with in VR.

Prompt: Rehabilitation games could be based on functional tasks – like preparing food in a virtual kitchen. They could also be more abstract, but incorporate important movements into the gameplay. Can you see advantages of either of these styles of games?

Follow-up: Think about these aspects of VR – escapism, competition, scores, progress, relaxation, cooperation. Do any of these appeal to you in a therapeutic context?

Follow-up: Sometimes VR games include a competitive aspect. What are your thoughts about having scoring systems, levels of difficulty, and competitions as a form of VR therapy?

~~Question 17: Sometimes therapy is administered in groups. Although the therapy an individual receives is specific to them, can you see VR being used in group situations?~~

~~What are the benefits to providing therapy in groups? Why do you think a group of patients using VR at the same time could be beneficial to them?~~

Question 18: Imagine yourself administering VR therapy - what kind of feedback would you want from a VR game?

Prompt: Is there real-time feedback you need to make sure that your patient is comfortable with the VR and/or benefitting from it?

Prompt: Would you require performance monitoring, some control over the delivery of the therapy, real-time feedback, measurement of progress of a patient?

Follow-up: How would that be beneficial to you?

~~Follow-up: And how do you think you could see this implemented in a VR game?~~

Conclude the session with a summary of the discussions, going over parts **1, 2, 3, and 4** of the focus group.

**Part 5:** Any other questions or ideas that the group wants to share.

[Ask the group if they would like to talk about anything else. Maybe there’s a point that they would like to make about something that’s already been spoken about, or something else entirely].

We would like to thank you for taking part in today’s focus group. Your contributions will be very useful for the next stages of this study, where we are going to use your responses to develop a suite of VR scenarios for upper limb rehabilitation.

Soon we will be looking to form an advisory panel of therapists to provide feedback on the design and development of the VR games. If you are interested in contributing to the project please get in touch via email.

[End the recording]

[end]
